# Radiofrequency Ablation for Adenomyosis

**DOI:** 10.3390/jcm12093069

**Published:** 2023-04-23

**Authors:** Ioannis Dedes, Georgios Kolovos, Arrigo Fruscalzo, David Toub, Cloé Vaineau, Susanne Lanz, Sara Imboden, Anis Feki, Michael D. Mueller

**Affiliations:** 1 Department of Obstetrics and Gynecology, University Hospital of Bern, University of Bern, 3010 Bern, Switzerland; georgios.kolovos@insel.ch (G.K.); cloe.vaineau@insel.ch (C.V.); susanne.lanz@insel.ch (S.L.); sara.imboden@insel.ch (S.I.); michel.mueller@insel.ch (M.D.M.); 2 Department of Gynecology and Obstetrics, University Hospital of Fribourg, 1752 Fribourg, Switzerland; arrigo.fruscalzo@h-fr.ch (A.F.); anis.feki@h-fr.ch (A.F.); 3 Gynesonics^®^, Redwood City, CA 94063, USA; dtoub@gynesonics.com

**Keywords:** radiofrequency ablation, adenomyosis, hyperthermia

## Abstract

Adenomyosis is a common benign gynecologic condition characterized by ectopic endometrial glands and stroma in the myometrium causing pain (dysmenorrhea) and abnormal uterine bleeding. New interventional techniques have been introduced over recent years. This study evaluates the treatment success and side effects of radiofrequency ablation. An electronic literature search in the PubMed, Scopus, and ScienceDirect databases was carried out on the outcomes of pain reduction and, secondarily, on abnormal uterine bleeding, reintervention, reproductive outcome, imaging outcome, and complications. There was a mean decrease in dysmenorrhea pain scores by −63.4 ± 9.0% at 12 months. Data on other outcome parameters were sparse. No major complications were reported. Radiofrequency ablation represents a promising minimally invasive and organ-preserving treatment in patients with symptomatic adenomyosis. It is associated with clinically meaningful improvement of adenomyosis-related pain in the short term.

## 1. Introduction

Adenomyosis is a chronic, hormone-driven, benign disease that is caused by ectopic endometrial glands and stroma leading to ill-defined lesions within the myometrium. These lesions can be focal or diffuse, are dispersed within the uterus, and are accompanied by hypertrophy and proliferation of neighboring myometrial cells.

The main symptoms of adenomyosis are pain (dysmenorrhea, pelvic pain) and abnormal uterine bleeding (AUB). It can also be associated with a lower clinical pregnancy rate and higher miscarriage rate and pregnancy complications [1,2] such as pre-eclampsia, preterm delivery, small for gestational age infancy, and post-partum hemorrhage.

The prevalence of adenomyosis is not well defined, as it can be difficult to diagnose and is often asymptomatic. For many years, adenomyosis remained a histopathological diagnosis made by the pathologist after examination of the hysterectomy specimen in perimenopausal women, mainly those with bleeding disorders. The estimated prevalence among hysterectomy patients ranges from 8.8 to 61.5%. The great variation in the prevalence can be attributed to the difficulty of histopathological diagnosis, as the disease might present to different extents: Adenomyosis can appear as a solitary unifocal lesions on a normal-sized uterus or as diffuse, globular enlargement of multiple weeks-size of the uterus through a diffuse infiltration of the uterine wall. Another rather rare subtype of adenomyosis is the “chimera” between uterine fibroids and adenomyosis, called adenomyoma. If only focal disease is present, it can be easily missed in histopathological examination if multiple sections of the hysterectomy specimen are not being carried out.

With the improvement of imaging techniques such as transvaginal ultrasound and magnetic resonance imaging, the diagnosis can nowadays more easily be established “in vivo”. Better imaging techniques thus lead to a shift in the age profile of patients diagnosed with adenomyosis towards women of childbearing age. It is estimated to affect up to 15–20% of women of reproductive age, and it commonly coincides with endometriosis and uterine fibroids [3].

In cases where adenomyosis and endometriosis are both present, endometriosis is associated with a greater extent of disease, classified as Stage III–IV by the American Society of Reproductive Medicine’s revised staging system for endometriosis (rASRM): approximately 50% of women with deep infiltrating endometriosis suffer from adenomyosis [4], and 80% of infertile women with proven endometriosis have adenomyosis [5,6]. Furthermore, the presence of adenomyosis may be one of the main factors in cases of symptom persistence [4,7,8] and in reduced incidence of pregnancy [9] after conservative endometriosis surgery.

Unlike endometriosis, no drug is currently labelled for the treatment of adenomyosis and there are no specific guidelines on its management. Symptoms can be controlled by hormonal treatment. While Dienogest is highly effective in endometriosis, it is unable to control abnormal uterine bleeding (AUB) in adenomyosis [10]. Levonorgestrel IUDs, which are effective in improving both symptoms (pain, AUB), are limited to small uteri due to a higher expulsion rate reported in larger adenomyotic uteri of up to 150 mL [10,11].

For patients with treatment-refractory or severe symptomatology, hysterectomy is considered the surgical standard treatment. In cases in which organ preservation is warranted, a spectrum of interventional procedures, such as uterine artery embolization (UAE), high-intensity focused ultrasound (HIF), and uterine-sparing surgery, which can include uterine artery ligation and adenomyosis excision, have been proposed. Surgical resection of diffuse and focal adenomyosis—excluding adenomyomas, a subtype of focal adenomyosis—is associated with significant perioperative risks and requires a highly elaborate surgical skill set. Combining endometriosis and adenomyosis surgery thus increases perioperative morbidity and, in particular, the risk of uterine rupture during pregnancy [12,13,14].

Radiofrequency ablation (RFA) belongs to the broader family of hyperthermic treatments, which includes treatments that utilize energy sources such as HIFU and percutaneous microwave ablation (PMWA). The delivery of radiofrequency energy has become an established treatment for uterine fibroids [15]. RFA involves the use of electrodes, which are directly inserted into the target lesion under ultrasound guidance. The electrodes generate heat energy via a high-frequency alternating electrical current (which produces friction) and induce thermal fixation and coagulative necrosis in the target lesion, which preserves the cellular architecture [16]. Secondary to the effects in the target lesion, there are also changes in the periphery [17]. These changes further cause uterus volume shrinkage through ablation-induced tissue contraction and dehydration.

These thermal effects also induce changes on a cytomolecular level, with decreased expression of estrogen (ER) and progesterone receptors (PR), and on the genetic level, with altered ER/PR and survivin genetic expression [17].

In this review, we evaluated outcomes after RFA in women with symptomatic adenomyosis.

## 2. Materials and Methods

This systematic review was conducted based on the “Preferred Reporting Items for Systematic Reviews and Meta-Analyses” (PRISMA) guidelines and selection criteria (Supplementary File S1–S3) [18]. The review protocol was registered in the PROSPERO online database (CRD 4202235126).

**Figure 1:**
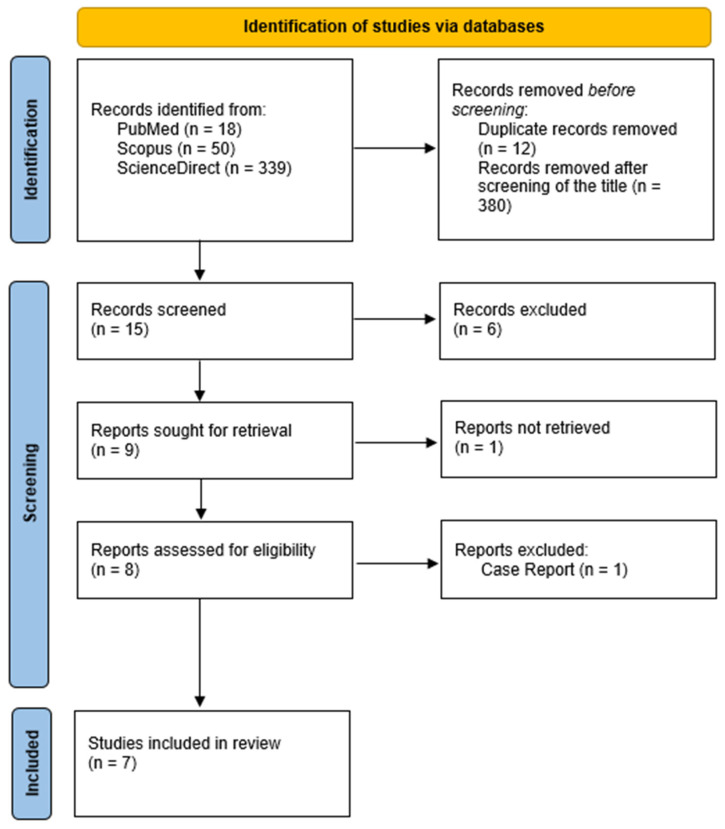
Flowchart for study selection according to the PRISMA guidelines.

Reports of women who underwent RFA for symptomatic adenomyosis were selected. The literature search for eligible studies was conducted in the electronic databases of PubMed, Scopus, and ScienceDirect, as shown in the flow chart (Figure 1). The research algorithm included the words “Radiofrequency Ablation” and “Adenomyosis”. Studies until 1 May 2022 in the English language were included. Study-type selection included randomized controlled trials (RCTs), cohort studies, and cases series, both retrospective and prospective. Two investigators (ID, GK) completed the main search independently, and any discrepancy was resolved after the main research was conducted.

Inclusion and exclusion criteria were established before the literature search, as part of the protocol declaration. The following inclusion criteria were applied: (1) epidemiological data such as age, duration, and severity of symptoms; (2) data regarding the adenomyosis diagnostic tool; and (3) data regarding the location and type of adenomyosis. Exclusion criteria consisted of the following: (1) data from sources other than original full publications (reviews, abstracts, oral presentations, national or local health statistics); and (2) studies not in the English language.

Data extraction articles and published abstracts were analyzed, and the following data were extracted: study design, year of publication, number of patients, mean age, mean uterine volume, average volume of lesion, and diagnostic modality of adenomyosis (MRI, ultrasound, biopsy). The primary outcome was reduction in pain according to the Visual Analog Scale and Visual Reporting System (VAS/VRS) and the Symptom Severity Score (SSS). The secondary outcomes were a reduction in abnormal uterine bleeding (AUB) as means of PBAC, a decrease in uterus and lesion size, complications, subsequent management, and reproductive outcome.

Because there is currently only one randomized controlled trial for RFA, and because of different follow-up times and heterogeneous endpoints, no meta-analysis was conducted. The different Pain Score Scales, (VAS 0–10, VRS 0–5, PAIN 0–20) were converted: VRS 0–5 was multiplied by two and PAIN 0–20 was divided by two, resulting in a “harmonized” scale of 0–10. For the adjusted pain score of 0–10, Symptom Severity Score (SSS), and morphological changes, the weighted means and weighted standard deviation at 12 months were calculated using R-Studio (RStudio Team (2020). RStudio: Integrated Development for R. RStudio, PBC, Boston, MA, USA) Weighted mean was only calculated on other parameters if they included >50% of subjects compared to the overall study population.

Because there was only one Pictorial Blood Loss Assessment Chart (PBAC) the reduction in patient-reported bleeding scores and AUB were presented descriptively. Reinterventions could not be pooled because of different time points of follow-up and a high number of patients lost to follow-up in the long-term studies; therefore, a descriptive presentation of the study results was performed. Narrative synthesis was performed for AUB, reproductive outcome, and complications. Results are presented as percentage change to baseline values.

The National Institutes of Health (NIH) quality assessment tool (https://www.nhlbi.nih.gov/health-topics/study-quality-assessment-tools, accessed on 10 July 2022) was used to describe the quality of the included studies (Supplementary File S1–S3). No ethics board approval was requested, as the data were extracted from published papers. After screening the titles and removing duplicates, the literature search resulted in 15 records. The abstracts of these records were further evaluated for eligibility. We excluded 8 articles that did not include any outcome measure of interest. One study did not provide a full text and was, therefore, excluded.

## 3. Results

Seven studies were included in this review, corresponding to 396 patients who underwent RFA treatment for symptomatic adenomyosis. The majority of these studies (5) were retrospective. There were two cohort studies [19,20] and one prospective randomized, parallel, controlled clinical trial [21]. The quality of the included studies ranged between good and poor. (Table 1) The mean loss to follow up was 12.5 ± 13.4% (Supplementary Table S1).

The mean age was 39.0 ± 2.3 (35.6–43) years. BMI (kg/m^2^) was available in four out of seven studies, and the mean was 22.4 ± 0.4 kg/m^2^. The predominant type of adenomyosis was of focal 75 ± 12.7%. Data on lesion and uterus volume were incomplete, with only four out of seven studies providing lesion volume data and three out of seven studies providing uterus volume data. The volume of focal lesions ranged between 25.5 and 71.7 cm^3^. Uterus volume ranged between 238.3 and 307 cm^3^ (Supplementary Tables S2 and S4).

Each RFA procedure was performed under sonographic guidance. The access of the electrode(s) to the uterus was transcervical in four studies, laparoscopic in two studies [19,22], and percutaneous in one study [21] (Supplementary Table S2).

Adenomyosis was diagnosed in all cases using transvaginal ultrasound and, in two studies, MRI was also used [21,23]. Only four studies referred to specific sonographic parameters applied for the diagnosis of adenomyosis, and only one for MRI. Confirmative core needle biopsy (CNB) of adenomyosis was performed in three studies [22,23,24] (Supplementary Table S2).

Reporting on concurrent endometriosis was available in five studies and was considered an exclusion criterion in four of them [21,22,24,25]. Stepniewska, A.K., et al. included patients with endometriosis who had undergone concurrent endometriosis surgery [19]. Two studies did not report the presence of endometriosis (Supplementary Table S2).

Uterine fibroids were excluded in five studies [19,21,22,23,25], whereas, in the remaining two studies, there was no information available on the presence on fibroids. One study reported pretreatment with a GnRH agonist 1–6 months prior to RFA in cases with diffuse adenomyosis and a uterine size over 14 weeks of gestation [24]. Another study combined RFA with placement of a levonorgestrel-containing IUD, of which 59.4% occurred immediately after RFA and the remaining occurred in the postoperative course up to 6 months after ablation [25]. The preoperative GnRH was intended to reduce the volume of the adenomyosis lesion and therefore facilitate RFA, whereas the postoperative levonorgestrel IUD placement was used to prevent recurrence. Because of the impact of postoperative IUD insertion on the reintervention-rate and imaging outcomes as stated by the authors, the study was excluded in the evaluation of those outcomes (Supplementary Table S2).

**Table 1:** Study Characteristics.

Study	No. of Patients	Loss to Follow Up	NIH Quality Assessment
Lin, X.L., 2020 [21]	65	0%	good
Nam, J.H., 2020 [24]	81	33.8%	fair
Hai, N., 2017 [23]	81	6.9%	good
Hai, N., 2021 [25]	64	12.3%	good
Scarperi, S., 2015 [22]	15	33%	fair
Stepniewska, A.K., 2022 [19]	60	0%	good
Dai Ti, S.A., 2018 [20]	30	n/a	poor

### 3.1. Symptoms

#### 3.1.1. Pain Scores

The main outcome parameter was menstrual pain scores expressed by a visual analog scale (VAS, in the range 0–10) and a visual reporting scale (VRS, in the ranges 0–5 and 0–20), which was reported in six out of the seven included studies. Four studies reported cure rates (complete relief, partial relief, no improvement). Follow-up at 12 months was available in all included studies. Two studies had a long-term follow-up of 56 months on average (Table 1).

The mean adjusted pain score prior to intervention was 7.7 ± 0.89 (Range 6.9–9.1), and this score decreased by −63.4 ± 9.0%, down to 2.7 ± 0.75 (Range 2.2–3.8), at 12 months. (n = 366) (Table 2).

#### 3.1.2. Symptom Severity Scores (SSS)

Symptom severity scores (SSS) were reported in three studies at 12 months (n = 210). SSS dropped by −59.1 ± 16.6%, from 34.9 ± 9.3 to 14.5 ± 2.2, at 12 months [22,23,25]. No improvement in dysmenorrhea was seen in 10.9 ± 6.9% (Range 3.3–19%) of cases [20,21,22] (n = 210) (Table 1, Supplementary Tables S3 and S4).

#### 3.1.3. Abnormal Uterine Bleeding (AUB)

Abnormal uterine bleeding was assessed in two studies, of which only one reported a −51.2% (n = 81) reduction via the Pictorial Blood Loss Assessment Chart at 12 months [24]. Stepniewska et al. reported an overall normalization of the bleeding pattern in 68.7% of cases with AUB (hypermenorrhea or continuous bleeding) and −64.3% in isolated adenomyosis (Supplementary Tables S3 and S4).

### 3.2. Rate of Reintervention

The overall reintervention rate after 12 months was reported to be 18.5% in one study [23] (total n = 81) consisting of repeat RFA in 3.7% (n = 3), levonorgestrel IUD insertion in 4.5% (n = 4), and hysterectomy in 9.9% (n = 8).

Another study [24] (total n = 81) with a longer follow-up showed a total reintervention rate of 39.5% (n = 32): 29.5% (n = 19) underwent repeat RFA at an average of 41.4 months (a range of 9–105 months), 4.5% (n = 4) underwent levonorgestrel IUD insertion at an average of 30.3 months (a range of 7–80 months), and 9.9% (n = 8) underwent hysterectomy at an average of 50.1 months (a range of 6–120 months).

The mean overall hysterectomy rate for treatment-failure was 10.8 ± 1.5%, calculated from 3 studies (n = 222) after 12 months. (The Study From Hai N, 2021, was excluded in this calculation because of the immediate and postoperative hormonal IUD-insertion over 6 months of 100%). Regarding the timing of hysterectomy, data was available from two studies: Stepniewska et al. reported a hysterectomy rate of 13% (n = 8) after 56 (16–78) months on average [19], and Nam HJ 2019 a rate of 9.9% at 50 (6–120) months.

Hysterectomy after RFA in combination with levonorgestrel IUD placement was reported in 1.6% of patients at 12 months [25] (Supplementary Table S4).

### 3.3. Reproductive Outcome

There were 31 patients who experienced 41 pregnancies overall (Supplementary Table S3). Only two of the included studies reported on pregnancies. The majority of information on pregnancies after RFA was derived from one study: Jang-Hyun Nam [24] reported 39 pregnancies in 29 patients in total, resulting in 24 deliveries (15 caesarean sections and 9 vaginal deliveries) in 22 patients. There were 12 spontaneous miscarriages and 3 artificial abortions. In addition, 25 of the 59 patients who attempted natural conception achieved 34 pregnancies, representing a clinical pregnancy rate of 42.7%. There were four out of twenty-two patients who attempted IVF, achieving five pregnancies. Overall, the clinical pregnancy rate was 35.8% and reached 50% after exclusion of those women who did not try to conceive and who inevitably or arbitrarily gave up attempting pregnancy. The total delivery rate was 66.7% (n = 24). There were three preterm births reported at 28, 32, and 35 weeks of gestation [19,24]. Most women (62.5%, n = 15) delivered by cesarean sections, and there were nine vaginal deliveries (37.5%). There were no uterine ruptures reported (Supplementary Table S4).

### 3.4. Imaging Outcome

Post-ablative volume reduction was reported in four studies, and this was assessed in three studies by ultrasound and in one by post-ablation MRI. There was a mean reduction in the adenomyosis lesion volume of −61.1 ± 20.5% [21,22,23], and total uterus volume of −46.0 ± 11.9% [21,22,23] was achieved after 12 months. The Study from Hai N, 2021 was excluded in this calculation because of the impact of IUD insertion on uterus volume. They achieved an overall reduction of −57% in uterine volume at 12 months.

One study reported complete disappearance in 20% of cases and a reduction in lesion size in 64% of cases, whereas lesions remained unchanged in 21% of cases [19] (Supplementary Table S4).

### 3.5. Complications

Minor complications were mostly vaginal discharge (a range of 1.2–100%), abdominal pain (13.3–26.5%), and low-grade fever. There was one expulsion of necrotic tissue, which was self-limiting, requiring no further intervention. As postoperative sequelae, intrauterine synechiae formation was reported in, on average, 3.6 ± 1.9% of cases [19,23,24] (Supplementary Tables S3 and S4).

## 4. Discussion

Uterine-sparing surgical techniques for adenomyosis have been proposed [13] but require highly complex surgical skills. Difficulty in correct reconstruction and hemostasis during uterine closure can lead to increased bleeding and a long operating time. Of particular concern is the risk of uterine rupture during pregnancy after surgery [12]. Moreover, as adenomyosis is characterized by the lack of clear demarcation between affected and healthy tissue, excision may result in incomplete removal or overly extensive removal (which can further increase the risk of uterine rupture in subsequent pregnancy). Simultaneous endometriosis and adenomyosis surgery is even more complicated.

As compared to excision, RFA is faster and less invasive/morbid. As compared to uterine artery embolization (UAE) and high-intensity focused ultrasound (HIFU), RFA can easily be incorporated into gynecological surgery. Furthermore, as RFA is guided by ultrasound, it allows the lesion to be more precisely targeted than surgery or UAE. Intraoperatively, a clear demarcation of healthy tissue from that affected by adenomyosis is often not present. Therefore, RFA treatment based on ultrasonographic mapping can lead to a more tailored approach and effectively spare uninvolved tissue.

Radiofrequency ablation appears to offer similar pain relief to that of conventional uterus-sparing surgeries. In a systematic review of conservative surgery for adenomyosis by Younes G. et al. 2018 [14] of mostly observational retrospective studies on 1398 patients, 50–94.7% of patients reported pain improvement and 25–80% had a reduction in menorrhagia within the first year. The recurrence rate was estimated to be between 9 and 19%.

The results of our review are also congruent with a recent meta-analysis on hyperthermic treatments (HIFU, PMWA, and RFA) by Liu L. et al. 2021. Their analysis on 15,908 patients derived from mostly single-arm clinical studies, consisting of mainly HIFU procedures (n = 15,123) and a few PMWA (n = 513) and RFA (n = 272). The relief rates of dysmenorrhea were 84.2% for HIFU, 89.7% for PMWA, and 89.2% for RFA [26]. Similar results have been shown for the clinical effectiveness of uterine artery embolization by de Bruijn A. et al. 2017. After data-synthesis of 1049 patients, they were able to show an improvement of symptoms in 83.1% of patients [27].

Our review revealed sparse and incomplete data on the effectiveness of RFA in relation to morphological changes in adenomyosis-associated AUB, uterus size and volume, and subsequent fertility and pregnancy. However, as Nam JH et al. [24] reported, RFA may be a feasible treatment for patients who want to maintain their fertility. Further data are needed to prove not just a possible improvement of fertility, but also safety during pregnancy. One should bear in mind the potential risks, such as uterine rupture, as there are two case reports after RFA in cases of uterine fibroids [28,29].

There are considerable limitations to this review that apply to clinical research on adenomyosis in general. First of all, there are no uniformly accepted or validated image-based criteria for adenomyosis—not to mention the inconsistency in histopathologic definitions [30]. Without harmonized classification systems for both ultrasound and MRI, difficulties in comparing different clinical studies on adenomyosis will remain. There are several ongoing attempts to achieve harmonization on the image-based criteria for the diagnosis of adenomyosis through ultrasound [31] and to develop core outcome sets and outcome definitions for studies on uterus-sparing treatments of adenomyosis [32].

In terms of this systematic review, imaging diagnostic criteria for adenomyosis were not just inconsistent, but also lacking: Only four out of seven included studies mentioned specific sonographic parameters applied for the diagnosis of adenomyosis, each of which referred to different classification criteria and studies. Comparisons among studies were further complicated by the heterogeneity in the variety of adenomyosis subtypes (i.e., diffuse vs. focal), medical pre-/post-treatment, scoring systems for symptom assessments, as well as different RFA-approaches.

Concerning the assessed outcome-parameters, there were few validated questionnaires and instruments for the evaluation of symptom outcomes, and clear definitions of the primary and secondary outcome, as well as definitions of clinical success, were lacking. Furthermore, long-term follow-up was only available in three studies and was accompanied by a high percentage of loss to follow-up.

From a methodological point of view, a high risk of selection bias was observed in the evaluated studies, as most of them had a retrospective design and were descriptive case series with a small number of patients assessed. All included articles had a high risk of bias in at least two domains, indicating a high risk of bias within this research. Other limitations consisted in the heterogeneity of the study population, additional hormonal treatment, and outcome parameters. For example, Nam J., 2020 et al. [24] reported on pretreatment with GnRH analogues in cases of large uteri over 14 weeks of gestation but did not specify the exact number of pretreated patients. Hai N. 2021, on the other hand, used a levonorgestrel IUD as an “adjuvant treatment” in all cases. The preoperative administration of GnRH analogues intended to reduce the volume of the adenomyosis lesion and therefore facilitate RFA, whereas the postoperative levonorgestrel IUD placement was used to prevent recurrence.

Another important limitation is the heterogeneous dealing with concurrent endometriosis, which may also influence the primary outcome, as it was excluded in 242 of 396 patients. Regarding the adenomyosis subtype, RFA was mainly used in focal lesions (Supplementary Table S2). Therefore, the results apply mainly for isolated and focal adenomyosis.

Despite those limitations, we conclude that RFA can be considered an effective treatment for symptomatic adenomyosis because of its effectivity in pain relief in women who wish to conserve their uterus. Further studies to consolidate these findings and to properly assess the effectivity in relation to other outcome parameters, such as AUB, quality of life, reproductive outcomes, etc., are needed.

## 5. Conclusions

Radiofrequency ablation represents a promising minimally invasive and organ-preserving treatment in patients with symptomatic adenomyosis. It is associated with clinically meaningful improvement of adenomyosis-related pain in the short term. Further studies are needed to corroborate a treatment effect beyond one-year follow-up and on other treatment outcome parameters, such as abnormal bleeding, quality of life, fertility, pregnancy outcome, or other clinical aspects.

## Figures and Tables

**Table 2:** Main outcome on pain.

Outcome Parameter	Weighted Mean	SD	No. of Patients
Pain Score, adj. (0–10)	−63.4%	±9.0%	n = 366
SSS ^1^	−59.1%	±16.6%	n = 210

^1^ Symptom Severity Score.

## Data Availability

The data presented in this study are available in Supplementary Tables S1–S4.

## Supplementary Materials

The following supporting information can be downloaded at: https://www.mdpi.com/2077-0383/12/9/3069/s1, Table S1: Study Design; Table S2: Study Properties; Table S3: Outcome 1; Table S4: Outcome 2. Supplementary File S1: NIH Quality Assessment Tool for Before-After (Pre-Post) Studies with No Control Group; Supplementary File S2: NIH Quality Assessment Tool for Cohort Studies; Supplementary File S3: NIH Quality Assessment Tool for Randomized Trials.
