# Supplementary material for: Radiofrequency Ablation for Adenomyosis
Source: J Clin Med. 2023 Apr 23;12(9):3069. doi: 10.3390/jcm12093069 (PMC10179480; doi:10.3390/jcm12093069)
Supplement: Supplementary file 1 [file jcm-12-03069-s001.zip › jcm-2232823-file S1.pdf]

File S1:

NIH Quality Assessment Tool for Before-After (Pre-Post) Studies With No Control Group

	1. Was the study question or objective clearly stated?	2. Were eligibility/selection criteria for the study population prespecified and clearly described?	3. Were the participants in the study representative of those who would be eligible for the test/service/intervention in the general or clinical population of interest?	4. Were all eligible participants that met the prespecified entry criteria enrolled?	5. Was the sample size sufficiently large to provide confidence in the findings?	6. Was the test/service/intervention clearly described and delivered consistently across the study population?	7. Were the outcome measures prespecified, clearly defined, valid, reliable, and assessed consistently across all study participants?	8. Were the people assessing the outcomes blinded to the participants' exposures/interventions?	9. Was the loss to follow-up after baseline 20% or less? Were those lost to follow-up accounted for in the analysis?	10. Did the statistical methods examine changes in outcome measures from before to after the intervention? Were statistical tests done that provided p values for the pre-to-post changes?	11. Were outcome measures of interest taken multiple times before the intervention and multiple times after the intervention?	12. If the intervention was conducted at a group level did th statistical analysis take into account the use of indivi data to determine effects at the group level?	**Overall (poor, fair, good)**
Nam JH, 2019	1	1	1	1	1	1	1	0	0	1	0	0	fair
Hai N, 2017	1	1	1	1	1	1	1	0	1	1	1	1	good
Hai N, 2021	1	1	1	1	1	1	1	0	1	1	0	0	good
Scarperi S, 2015	1	1	1	0	0	1	0	0	0	1	0	0	fair
Stepniewska AK 2022	1	1	1	1	1	1	1	0	1	1	0	0	good

[The National Institutes of Health (NIH) quality assessment tool (https://www.nhlbi.nih.gov/health-topics/study-quality-assessment-tools)](https://www.nhlbi.nih.gov/health-topics/study-quality-assessment-tools)
