# Supplementary material for: Radiofrequency Ablation for Adenomyosis
Source: J Clin Med. 2023 Apr 23;12(9):3069. doi: 10.3390/jcm12093069 (PMC10179480; doi:10.3390/jcm12093069)
Supplement: Supplementary file 1 [file jcm-12-03069-s001.zip › jcm-2232823-Table S1.pdf]

Supplementary Table S1: Study Design

Study Design/Properties								Epidimiological data		Follow up				Study Quality
Author	Study	Year	Country	Study Design			No of patients	mean age	BMI (kg/m2)	time (months)	loss to follow up (12 months)	Assessment		
												Modality	point of time (months)	NIH Rating
Lin XL, 2020	Comparison between microwave ablation and radiofrequency ablation for treating symptomatic uterine adenomyosis	10/2015 - 10/2017	China	Experimental (II)	single-center, randomized, parallel and controlled clinical trial	prospective	65	38.9 ± 5.0	22.0 ± 3.3	12	none	post ablation CEUS and cMRI at day3, SSS and VRS Scores thereafter inlcuding TVUS.	(day 3 MRI) 3,12	good
Nam JH., 2020	Pregnancy and symptomatic relief following ultrasound-guided transvaginal radiofrequency ablation in patients with adenomyosis	08/2004 - 11/2014	South Korea	descriptive (VI)	case series	retrospective	81	35.6 (26–44)	n/a	57.4 (3-129)	33.8% (26) on outcome symptoms. 5% (4) on outcome pregnancy	PBAC and PAIN Score	1,3,6,12,24,36,48	fair
Hai N, 2017	Ultrasound-guided transcervical radiofrequency ablation for symptomatic uterine adenomyosis	2013 - 10/2015	China	descriptive (VI)	case series	retrospective	81	40.0 ± 8.5	22.5 ± 2.4	12	6.9% (6/87)	post ablation CEUS (= NPV 70%) and then TVUS thereafter. VAS	1,6,12	good
Hai N, 2021	Ultrasound-guided transvaginal radiofrequency ablation combined with levonorgestrel-releasing intrauterine system for symptomatic uterine adenomyosis treatment	01/2013 - 01/2016	China	descriptive (VI)	case series	retrospective	64	39.2 ± 4.9	22.5 ± 2.9	12	12.3% (9/73)	post ablation CEUS (= NPV 70%) and then TVUS thereafter. Blood sampels at 3,6 months, VAS	3, 6,12, 24, 36	good
Scarperi S, 2015	Laparoscopic Radiofrequency Thermal Ablation for Uterine Adenomyosis	n/a	Italy	descriptive (VI)	case series	prospective	15	40.1 (34–46)	23.9	9 (3-12)	33% (5)	Ultrasound/VAS	3, 6, 9, 12	fair
Stepniewska AK, 2022	Heat can treat: long-term follow-up results after uterine-sparing treatment of adenomyosis with radiofrequency thermal ablation in 60 hysterectomy candidate patients	03/2011 - 06/2019	Italy	observational (III)	cohort study	retrospective	60	43 (38–50) ± 3.0	n/a	56 (10–115, ± 29)	none	Ultrasound/Scores	not specified	good
Sha A Dai Ti WFE, 2018	Clinical evaluation of three methods in the treatment of adenomyosis	01/2013 - 01/2016	China	observational (III)	cohort study	retrospective	30	37.33 ± 4.25	n/a	12	n/a	n/a	n/a	poor
	**Weighted Mean**							**39.0**	**22.4**	**27.8**	**12.5%**			
	**Weighted SD ±**							2.3	0.4	21.6	13.4%			
