# Supplementary material for: Radiofrequency Ablation for Adenomyosis
Source: J Clin Med. 2023 Apr 23;12(9):3069. doi: 10.3390/jcm12093069 (PMC10179480; doi:10.3390/jcm12093069)
Supplement: Supplementary file 1 [file jcm-12-03069-s001.zip › jcm-2232823-Table S2.pdf]

Supplementary Table S2: Study Properties

	**Diagnostic Modality**				**Adenomyosis Specifications**							**Procedure Specifications RFA**						**Accopanying disease**			
					Type			Localization				Access									
Author	MRI	Applied Criteria/Reference	TVUS	Applied Criteria/Reference	Focal	Diffuse	Adeno myoma	Anterior	Posterior	Lateral	Fundal/ Both	Laparoscopic	Transcervical	Percutaneous	Biopsy of Lesion	RFA margin	Pretreament	Endometriosis	concurrent endo. Surgery	history of endo. Surgery	Fibroids
Lin XL, 2020	1	n/a	1	n/a	n/a	n/a	n/a	51.0%	4.6%	44.6%	n/a	0	0	1	n/a	3-5mm (serosa and endometrium)	n/a	excluded (OMA)	0	0	excluded
Nam JH., 2020	0	n/a	1	"Enlarged globular uterus, myometrial cyst, linear myometrial striations, poor delineation of the endomyometrial junction and heterogeneous myometrium"	79%	21%	n/a	16.0%	63.0%	n/a	21.0%	0	1	0	CNB	10mm (serosa and endometrium)	GnRH Agonist (1-6 months) for Pt. With enlarged uterus >14 weeks of gestation	17.3% (14)	0	1	n/a
Hai N, 2017	1	Reinhold; Hum Reprod Update 1998	1	Fedele L, Am J Obstet Gynecol 1992	81.5% (66)	18.5% (15)	n/a	30.3%	57.6%	n/a	12.1%	0	1	0	CNB (uncertain cases)	discontinuation if 80–90% of the volumetric lesion turned hyperechogenic	n/a	excluded	0	0	excluded
Hai N, 2021	0	n/a	1	n/a	n/a	n/a	n/a	39.0%	66.2%	n/a	12.5%	0	1	0	n/a	discontinuation if 80–90% of the volumetric lesion turned hyperechogenic	n/a	n/a	0	0	excluded
Scarperi S, 2015	0	n/a	1	Abrao MS, Hum Reprod. 2007	100%	n/a	n/a	27.0%	53.0%	n/a	20.0%	1	0	0	CNB	n/a	n/a	excluded	1	0	excluded
Stepniewska AK, 2022	0	n/a	1	T Bosch Van den M; Ultrasound Obstet Gynecol. 2015	55% (33)	22% (13)	23% (14)	15.0%	58.0%		10.0%	1	0	0	n/a	n/a	n/a	65% (39)	1	1 (37% n=22)	excluded
Sha A Dai Ti WFE, 2018	0	n/a	1	n/a	n/a	n/a	n/a	n/a	n/a	n/a	n/a	0	1	0	n/a	n/a	n/a	n/a	0	0	n/a
	**Weighted Mean**				**75%**	**20%**		**30%**	**51%**		**15%**										
	**Weighted SD ±**				12.7%	1.5%		13.2%	21.8%		4.4%										

Studies:

Reinhold ; Imaging features of adenomyosis. Hum Reprod Update 1998

Fedele L, . Transvaginal ultrasonography in the differential diagnosis of adenomyoma versus leiomyoma. Am J Obstet Gynecol 1992;

Abrao MS; Comparison between clinical examination, transvag-inal sonography and magnetic resonance imaging for the diag-nosis of deep endometriosis. Hum Reprod. 2007

T Bosch Van den M; Terms, defnitions and measurements to describe sonographic features of myometrium and uterine masses: a consensus opinion from the morphological uterus sonographic assessment (MUSA) group Ultrasound Obstet Gynecol 2015

Abbreviations:

OMA: Ovarian Endometrioma

CNB: Core Needle Biopsy
