# Supplementary material for: Radiofrequency Ablation for Adenomyosis
Source: J Clin Med. 2023 Apr 23;12(9):3069. doi: 10.3390/jcm12093069 (PMC10179480; doi:10.3390/jcm12093069)
Supplement: Supplementary file 1 [file jcm-12-03069-s001.zip › jcm-2232823-Table S3.pdf]

Supplementary Table S3: Outcome 1

Author	No. Of Patients	**Adenomyosis Symptoms**						**AUB**		**Reinterventions**				**Complications**				**Reproductive Outcome**		
		Baseline			Outcome			Outcome		Total (%) (mean months)	Repeat RFA (mean months)	Hormonal treatment/IUD (mean months)	Hysterectomy (mean months)	Pain	Minor	Major	Synechia	total	in n patients	preterm deliver
		Dysmenorrhea	AUB	Both	Complete	Partial	No improvement	Persisting AUB	% Change											
Lin XL, 2020	65	81.5% (53)	n/a	n/a	49.3% (26)	43.1% (23)	7.6% (4)	n/a	n/a	n/a	n/a	n/a	n/a	abdominal pain	vaginal discharge low grade fever (<14d)	0	n/a	n/a	n/a	n/a
Nam JH., 2020	81	22.4% (13)	6.9% (4)	70.1% (41)	n/a	n/a	n/a	n/a	n/a	39.5% (n=32) (41.4, 9-105)	23.5% (n=19) (41.4, 9-105)	7.4% (n=6) (30.3, 7-80)	9.9% (n=8) (50.1, 6-120)	n/a	2 (vaginal discharge)	1 (expulsion necrotic tissue)	2.5% (n=2)	39	36% (n=29)	2 (32 and 28 weeks of gestation)
Hai N, 2017	81	92.6% (75)	n/a	n/a	n/a	n/a	18.5% (15)	n/a	n/a	18.5% (n=15)	3.7% (n=3)	4.9% (n=4)	9.9% (n=8)	n/a	n/a	n/a	2.5% (n=2)	n/a	n/a	n/a
Hai N, 2021	64	79.7% (51)	65.6% (42)	n/a	n/a	n/a	n/a	n/a	n/a	n/a	n/a	100%	1.6% (n=1)	26.5% (17)	100% vaginal discharge; (2 vaginal dischagre>14d with antibiotic treatment)	n/a	n/a	n/a	n/a	n/a
Scarperi S, 2015	15	100%	n/a	n/a	20% (1)	80% (8)	0	n/a	n/a	n/a	n/a	n/a	n/a	13.3% (2)	0	0	n/a	n/a	n/a	n/a
Stepniewska AK, 2022	60	53% (32)	52% (31)	45% (27)	n/a	n/a	n/a	36% (10/31)	-68.7%	n/a	n/a	20% (n=12)	13.3% (n=8) (42, 16-78)	n/a	1.7% (fever, treated with antibiotics)	n/a	6.7% (4)	2	2	1 (35 weeks of gestation)
		63% (10/16 amenorrhoea excluded)	67% (14/21)	n/a	n/a	n/a	19% (3/16)	24% (5/14)	-64.3%											
Sha A Dai Ti WFE, 2018	30	n/a	n/a	n/a	67% (20)	30% (9)	3% (1)	n/a	n/a	n/a	n/a	n/a	n/a	oc-casionally	fever (oc-casionally)	n/a	n/a	n/a	n/a	n/a
	**Weighted Mean**	**69.3%**	**24.0%**	**59.4%**	**50.1%**	**44.6%**	**10.9%**					**9.9%**	10.8%							
	**Weighted SD ±**	26.4%	31.3%	12.5%	14.3%	15.2%	6.9%					6.3%	1.5%							

*p<0.05 vs. previos assessment
