# Supplementary material for: Radiofrequency Ablation for Adenomyosis
Source: J Clin Med. 2023 Apr 23;12(9):3069. doi: 10.3390/jcm12093069 (PMC10179480; doi:10.3390/jcm12093069)
Supplement: Supplementary file 1 [file jcm-12-03069-s001.zip › jcm-2232823-Table S4.pdf]

Supplementary Table S4: Outcome 2

**Pain Scores (VRS/VAS/PAIN)**							**Symptom Severity Score (SSS)**				**Pictoral Bleeding Assessment Chart (PBAC)**							**Morphologic Changes**					
	Outcome							Outcome				Outcome						Basline	Outcome		Baseline	Outcome	
Baseline	1 month	3 months	6 months	12 months	%	12+ months	Basline	12 months	%	12+ months	Baseline	1 month	3 months	6 months	12 months	%	12+ months	mean Vol. of lesion (cm3)	(cm3) at 12 months	%	mean Vol. of uterus (cm3)	(cm3) at 12 months	%
3.91 ± 0.98 (VRS 0-5)	n/a	n/a	n/a	1.92 ± 0.79*	**-50.9%**	n/a	21.8 ± 5.5	16.4 ± 4.8	-34.8%	n/a	n/a	n/a	n/a	n/a	n/a	n/a	n/a	71.1 ± 57.3	n/a	-67.3%	238.3 ± 129.8	n/a	-47.6%
18.1 (PAIN 0–20)	13.3* ± 12.5	13.7* ± 13.0	13.7* ± 10.6	5.62 (n=26)	**-69.0%**	8.87; 24 months (n=23) 7.8; 36 months (n=15); 1.29; 48 months (n=7)	n/a	n/a	n/a	n/a	234 (19–815)	104.3* ± 178.6	118.4* ± 194.1	120.0* ± 139.3	111.58	-52.3%	131.0; 24 months, (n=23) 248.1; 36months, (n=15) 106.4; 48 months (n=7)	n/a	n/a	n/a	n/a	n/a	n/a
6.9±0.8 (VAS 0-10)	n/a	n/a	n/a	1.9 ±1.2*	**-72.5%**	n/a	44.17 ± 11.3	11.85 ± 8.24	-73.2%	n/a	n/a	n/a	n/a	n/a	n/a	n/a	n/a	58.4 ±35.6	29.6 ± 16.2	-41.2%	260.9 ± 149.3	153.4 ± 93.7*	-41.2%
7.2 ± 1.9 (VAS 0-10)	n/a	3.5 ± 1.3*	3.0 ± 1.1*	2.2 ± 0.9*	**-69.4%**	2.3 ± 0.8; 24 months 2.3 ± 1.0; 36 months	36.4 ± 13.6	16.1 ± 4.1*	-65.8%	17.6 ± 5.6* 24 months 17.5 ± 4.3* 36 months	n/a	n/a	n/a	n/a	n/a	n/a	n/a	n/a	n/a	n/a	307.0 ± 183.1	132.2 ± 60.2*	excluded (-57.0%)
9.1 (VAS 0-10)	n/a	5.4* (n=15)	3.8* (n=13)	2.6 (n=10)	**-71.4%**	n/a	n/a	n/a	n/a	n/a	n/a	n/a	n/a	n/a	n/a	n/a	n/a	60 (18-128)	20.8	-63.7%	n/a	n/a	-65.3%
7.4 (VAS 0-10)	n/a	n/a	n/a	3.3* (Δ 4.1)	**-55.4%**	n/a	n/a	n/a	n/a	n/a	n/a	n/a	n/a	n/a	n/a	n/a	n/a	n/a	Disappearance in 11 (20%) Reduction in 36 (64%) No Change in 12 (21%) (n=56)	n/a	n/a	n/a	n/a
7.1 (VAS 0-10)	n/a	n/a	n/a	4.0* (Δ 2.73)	**-43.7%**																		
n/a	n/a	n/a	n/a	n/a	**n/a**	n/a	n/a	n/a	n/a	n/a	n/a	n/a	n/a	n/a	n/a	n/a	n/a	25.50 (44.18)	0 (3.28)	-100.0%	n/a	n/a	n/a
**7.7 (adj)**				**2.7 (adj)**	**-63.4%**		**34.9**	**14.5**	**-59.1%**											**-61.1%**			**-46.0%**
0.89				0.75	9.0%		9.3	2.2	16.6%											20.5%			11.9%
